# Detection Limits of Blood Metabolites at Physiological Concentrations Using Benchtop H NMR

**DOI:** 10.1002/nbm.70215

**Published:** 2025-12-23

**Authors:** Alexander D. Hill, Gil Travish, Marie Phelan, Morgan Hayward, Carsten P. Welsch

**Affiliations:** ^1^ Department of Physics University of Liverpool Liverpool UK; ^2^ The Cockcroft Institute Sci‐Tech Daresbury Warrington UK; ^3^ ViBo Health Inc. Los Alamos New Mexico USA; ^4^ Department of Biochemistry and Systems Biology, Institute of Systems Molecular and Integrative Biology University of Liverpool Liverpool UK; ^5^ Department of Molecular and Cell Biology, Leicester Institute of Structural and Chemical Biology University of Leicester Leicester UK

**Keywords:** applications, low‐field NMR, metabolic tracking, metabolomics, methods and engineering, MRS, personalised healthcare, postacquisition processing

## Abstract

Commercial low‐field (LF) magnetic resonance spectroscopy (MRS) offers a route to rapid and repeated in vivo metabolite tracking; however, its sensitivity and interpretability at physiological concentrations remain underexplored. Here, we evaluate the performance of an 80‐MHz benchtop nuclear magnetic resonance (NMR) spectrometer (Bruker Fourier 80) across several key blood metabolites at physiological concentrations, ranging between 0.05 and 10.0 mmol/L. We characterise the relationship between metabolite concentration, acquisition time and signal‐to‐noise ratio (SNR) for multiple pulse sequences and assess how the choice of SNR definition influences reported detection and quantification thresholds. Metabolites present at millimolar levels, such as glucose and lactate, were readily detectable within 20 s, with the water‐suppressing wet pulse sequence yielding the highest SNR at fixed acquisition time. Concentration differences were also readily distinguishable. In contrast, submillimolar metabolites such as citrate require over 4 min to reach conventional detection thresholds, constraining their applicability to rapid metabolite tracking via MRS. To address interpretability in low‐SNR data, we introduce a template‐fitting approach based on simulated standards from CcpNMR AnalysisAssign, which stabilised relative metabolite quantification under low‐SNR conditions. These results establish quantitative benchmarks for LF NMR metabolite detection and demonstrate how simulation‐assisted analysis can extend its utility. These findings inform both the selection of target metabolites and optimisation strategies for emerging commercial in vivo MRS devices, including the DigiScan^TM^ finger‐scanner, supporting their development as accessible tools for real‐time metabolic tracking in personalised healthcare.

## Graphical abstract

We demonstrate rapid, sensitive detection of key blood metabolites using an 80 MHz low‐field NMR spectrometer, benchmarking signal‐to‐noise ratios and acquisition efficiency across experimental designs. Millimolar metabolites such as glucose and lactate are reliably detected and quantified within seconds, while simulation‐assisted spectral fitting further improved quantification under low‐SNR conditions. These findings guide translational applications in diagnostic biomarker selection and instrument optimization for future real‐time in vivo metabolic tracking for personalized health.

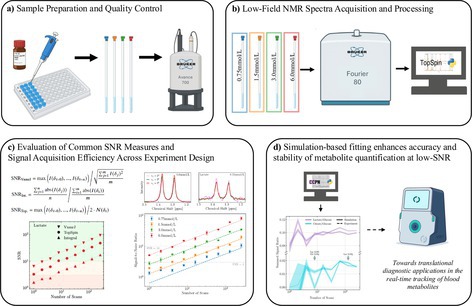

**Abbreviations**
1D: one‐dimensional
CcpNMR: collaborative computational project for NMR
D
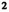
O
deuterium oxide (deuterated water/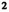
H
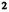
O)
ESI: electronic supplementary material
GISSMO: guided ideographic spin system model optimization
HMDB: human metabolome database
LF: low‐field
LOD: limit of detection
LOQ: limit of quantification
MRS: magnetic resonance spectroscopy
NMR: nuclear magnetic resonance
NOESY: nuclear overhauser effect spectroscopy
RF: radiofrequency
SNR: signal‐to‐noise ratio
TSP: trimethylsilylpropanoic acid
WET: water suppression enhanced through T1

## 1 Introduction

Healthcare provision has traditionally been guided by population studies, which define expected normal ranges for various diagnostics and inform the development of standardised care pathways. Personalised healthcare has recently emerged as a means of tailoring treatment to account for an individual's unique physiology. This has been driven by advances in fields such as genomics, proteomics, metabolomics and artificial intelligence. In its ideal form, personalised health integrates diverse, information‐rich datasets to detect deviation from an individual's baseline, support informed decision‐making and ultimately predict and prevent future health issues [1, 2, 3]. Parallel to this, consumer‐grade wearable devices have increased in popularity, providing users with real‐time, actionable health data that support healthy behaviours and help manage chronic conditions [4, 5, 6].

Metabolomics is of increasing interest in personalised healthcare, as metabolites are intermediates and end products of biochemical pathways and therefore reflect a broad spectrum of physiological states [7, 8, 9]. A common method for identifying and quantifying metabolites in biofluids is proton nuclear magnetic resonance (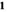
H NMR), which exploits the magnetic properties of hydrogen nuclei to reveal molecular structures. NMR‐based metabolomics studies typically employ high‐field (HF) spectrometers, whose strong and highly uniform magnetic fields enable the acquisition of high‐resolution and high‐sensitivity spectra [10, 11, 12, 13, 14]. HF spectrometers rely on cryogenically cooled superconducting magnets, whereas low‐field (LF) spectrometers use permanent magnets [15, 16, 17, 18]. Often referred to as ‘benchtop’ devices due to their compact size, LF spectrometers offer key advantages despite lower sensitivity and resolution. They are cryogen‐free, more affordable and require less specialist training to operate. As a result, benchtop NMR devices are increasingly used in both research and applied settings across public and private sectors [19, 20, 21, 22, 23, 24, 25, 26].

ViBo Health^1^ is developing DigiScan^TM^, a personalised healthcare device that applies the accessibility advantages of benchtop NMR technology to enable real‐time, in vivo metabolite tracking. The device utilises magnetic resonance spectroscopy (MRS) in a tabletop format, translating the compact, user‐friendly design principles of benchtop spectrometers to direct tissue analysis. While current MRS‐based metabolite tracking uses whole‐body or preclinical scanners [27, 28, 29]., DigiScan is designed as a finger‐scanning device, functionally analogous to benchtop NMR spectrometers, which analyse samples placed within a compact bore. In contrast to wearable technologies that monitor metabolites through sweat [30]. or interstitial fluid [31, 32]., DigiScan aims to enable targeted, noninvasive analysis of blood, the fluid most closely linked with systemic metabolism [33, 34, 35].

DigiScan and all NMR‐based technologies are affected by the need for metabolites to be present above a certain concentration threshold to be reliably detected. As typical blood metabolite concentrations vary widely, this naturally limits which molecules may be targeted, particularly at low magnetic field strengths where sensitivity is reduced. Theoretically in laboratory settings, a spectrum's signal‐to‐noise ratio (SNR) can be increased arbitrarily by conducting repeated measurements over an extended acquisition time. In contrast, consumer‐facing devices must emphasise rapid and reliable data collection, leading to practical constraints on acquisition time. It is therefore essential to identify which metabolites are quickly detectable at LF. Moreover, to enable meaningful health monitoring, a scanner must be capable of distinguishing physiologically relevant fluctuations in metabolite concentrations. Understanding these limitations is critical to guiding both technical development and the selection of suitable metabolic biomarkers.

In this work, we aim to determine which of several key metabolites may be quickly identified by DigiScan and at what physiological concentrations. We prepare individual and mixed samples of glucose, lactate and citrate across a range of physiologically relevant concentrations and acquire NMR spectra using a commercial Bruker Fourier 80 spectrometer, which operates at a magnetic field strength comparable to that of DigiScan. We calculate the SNRs of the metabolite standards and mixed sample spectra as a function of concentration and NMR experiment design and discuss the implications for DigiScan. We also explore the use of simulation‐based template fitting as a means of increasing confidence in metabolite measures in noisy spectra. Section 2 outlines the materials and methods used in this study; Section 3 presents our findings, and Section 4 provides a summary and key conclusions. The Supporting Information (SI) presents additional methodology and findings associated with this work.

## 2 Materials and Methods

This study assesses the ability of LF NMR spectrometers to quickly detect key metabolites found in blood at physiological concentrations. Section 2.1 describes the sample preparation, Section 2.2 covers the spectrometer used and the initial signal processing, Section 2.3 describes the simulations used to predict spectral feature locations and guide template fitting and Section 2.4 details the numerical methods for SNR computation.

### 2.1 Sample Preparation

Powdered forms of D‐Glucose, Sodium L‐Lactate and Citric Acid were purchased from Sigma‐Aldrich Ltd. (now Merck, Gillingham, UK) and dissolved in 99.9% 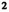
H
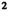
O containing 100 mmol/L sodium phosphate buffer^2^ (Thermo Fisher Scientific) for pH stabilisation and d4 selectively deuterated 100 μmol/L trimethylsilylpropanoic acid (TSP; Sigma‐Aldrich Ltd.) as a 0‐ppm chemical shift reference. The final samples were 600 μL contained within 5‐mm outer diameter NMR tubes. Both single‐metabolite and mixed samples were prepared. 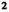
H
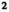
O was chosen as the solvent to minimise the potential impact of spectral overlap and receiver saturation due to the presence of 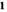
H
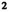
O water, thereby representing a ‘best‐case scenario’ for a future commercial device aiming to effectively suppress the water signal.

The single‐metabolite standards were prepared at four concentrations, approximately spanning abnormal and normal ranges reported in blood on the Human Metabolome Database HMDB [36]., with each concentration doubling across the series. Glucose samples were prepared at 1.25, 2.5, 5.0 and 10.0 mmol/L; lactate at 0.75, 1.5, 3.0 and 6.0 mmol/L; and citric acid at 0.05, 0.1, 0.2 and 0.4 mmol/L. For each metabolite and concentration pairing, three replicate samples were prepared to ensure reproducibility in both sample preparation and spectrometer response. Validation results are provided in Section 1 of the SI and shown in Figure S1. Citric acid samples are interchangeably referred to as citrate in this work.

A mixed sample was also prepared, containing 10 mmol/L glucose, 2 mmol/L lactate and 0.2 mmol/L citric acid. The same buffer composition as the single‐metabolite standards were used. This mixture was used primarily to assess suitability of dynamic range within mixture analysis, demonstrating spectrometer performance and also providing a comparison of relative abundances with SNR measures and further data processing methods.

### 2.2 Spectra Acquisition and Processing

We used the Bruker Fourier 80 spectrometers (Bruker Biospin, Coventry UK and University of Liverpool, Liverpool UK) operating at transmitter frequencies of 80.1MHz and compared the performance of three pulse sequences: zg, zg30 and wet. The zg sequence is a standard 1D experiment comprising a 90° pulse to rotate nuclear spins into the transverse plane, while zg30employs a 30° pulse. This returns a weaker signal per scan but enables shorter relaxation delays, which can improve SNR per unit time. The wet sequence suppresses the residual 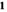
H
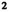
O signal through a combination of selective presaturation and dephasing pulses, which allows for higher receiver gain and potentially enhances the detectability of metabolite signals [37]. However, potential limitations of the wet sequence include the sensitivity of solvent frequency calibration to drift in sample temperature and pH, partial attenuation of resonances near the water peak and relaxation‐dependent biases that may affect quantitation.

Initial processing of spectral data occurred within topspin (4.3.0) and included the application of an exponential window function corresponding to a line broadening of 0.3 Hz, Fourier transformation, automated phasing and spectral alignment to the internal standard, TSP (set at 0.00 ppm). topspin automatically scales signal intensity to the highest peak height. Separately, in postprocessing we scale signal intensities according to the integrated intensity of TSP, which is held to be of fixed concentration across samples. In this publication, these sets of spectra are referred to as ‘unscaled’ and ‘scaled’, respectively.

In addition to metabolite concentration and experiment design, NMR signal intensity and detection sensitivity are further influenced by several hardware‐dependent factors, including the efficiency of the detection coil, the filling factor between sample and coil volumes and the performance of the associated RF electronics. While comparison between commercial systems and prototype hardware, such as the Bruker Fourier 80 and DigiScan devices, therefore requires careful consideration of these factors, the fundamental relationships between metabolite concentration, acquisition time and SNR remain applicable across platforms and provide a framework for experimental design. Commercial systems, in which hardware and electronic parameters have been optimised, further serve as a useful benchmark for spectrometer performance.

Further details of the spectrometer configurations, spectra acquisition and initial data processing, such as baseline correction and alignment, may be found in Section 2 of SI. Raw data, including acquisition and processing parameters, are available on the European Bioinformatics Institute metabolomics public repository (Accession number MTBLS6262).

### 2.3 Simulated Metabolite Standards

We use simulated metabolite spectra to provide a theoretical reference for interpreting experimental data. These were created using CcpNMR's AnalysisAssign package [38, 39]. alongside tools from AnalysisMetabolomics. This software contains tools for the generation of individual or combined metabolites spectra at a specified magnetic field strength.

The simulated metabolite spectra generated in AnalysisAssign rely on theoretical models of spin‐coupling specified by the Guided Ideographic Spin System Model Optimisation [40]. (GISSMO) library, with experimentally validated values from the small molecule spectra available in the Biological Magnetic Resonance Data Bank [41]. Theoretical spin‐coupling calculations generate a set of energy and intensity value pairs, which are rendered as peaks using Lorentzian line‐shapes centred at each energy value with amplitudes proportional to the respective intensity. These peak curves are summed to produce a simulated spectrum for each metabolite, which can then be combined with others to model mixtures.

Simulated standards enable flexible and efficient metabolite profiling by allowing adjustment of chemical shift values to accommodate for variations in experimental conditions, thus eliminating the need for extensive collection of NMR metabolite standards. Importantly, these simulations incorporate strong coupling effects, which are especially pronounced in LF NMR and therefore critical for accurate spectral modelling.

#### 2.3.1 Determination of the Metabolite Bounds

**FIGURE 1:**
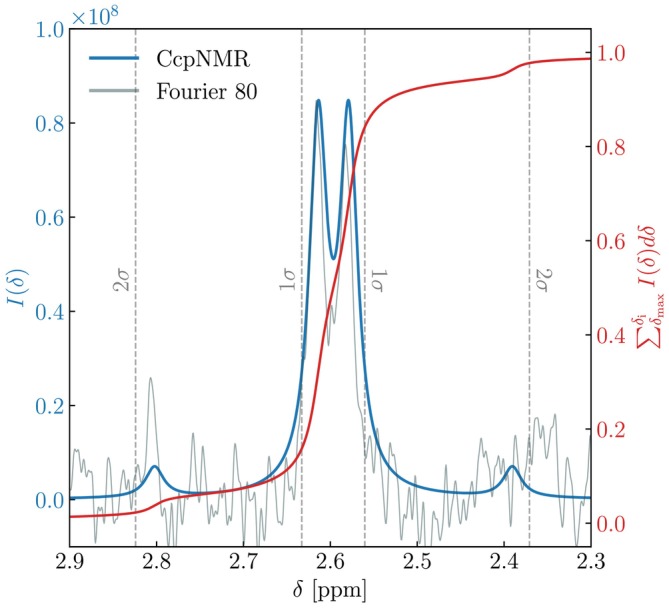
Example usage of CcpNMR AnalysisAssign to define the expected ppm bounds of a metabolite's NMR spectrum. The blue curve shows the simulated spectrum of citrate at 80 MHz. The grey background data depict an experimentally acquired spectrum of citrate from a Bruker Fourier 80 benchtop spectrometer, shown here for reference. The red curve shows the cumulative sum of the theoretical signal intensity, normalised to one and integrated from high to low ppm. The centre of the spectrum is determined to be ∼2.6 ppm, the value at which the cumulative sum is 0.5. Dashed lines indicate regions containing 1σ (∼68%) and 2σ (∼95%) of the total signal, defined as the ppm values where the cumulative sum reaches approximately [0.159, 0.841] and [0.023, 0.977], respectively. The expected bounds are taken as the region spanning 2σ.

Expected chemical shifts for each metabolite were estimated from simulated 80 MHz spectra, with peak boundaries defined as the region containing  95% of signal intensity. The application of this method with respect to citrate is illustrated in Figure 1; further details of this methodology and its application to glucose and lactate are provided in Section 3 of the SI and displayed in Figure S2.

The calculated bounds were as follows (in ppm): glucose [3.19, 3.98], lactate [1.17, 1.50] and citrate [2.37, 2.82] ppm. For citrate, the full spectral width of the simulated spectrum was used in the analysis. For lactate, we restricted our analysis to the prominent doublet at 1.35 ppm and excluded the weaker signal at ∼4.1 ppm, while for glucose, we restricted the bounds to exclude the anomeric peaks between 4 and 5.5 ppm. This was done in order to avoid spectral overlap with the residual 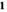
H
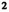
O water signal.

### 2.4 Computation of the SNR

We used SNR as a measure of the strength of detection for each metabolite. Here *signal* and *noise* are computed as the mean absolute intensities within designated regions

(1) $$\text{SNR}=\frac{\sum_{j=1}^{n}\text{abs}(I(\delta_{j}))}{n}/\frac{\sum_{i=1}^{m}\text{abs}(I(\delta_{i}))}{m},$$

where n and m are the number of data points in the *signal* and *noise* regions, respectively, and $I(\delta_{x})$ is the intensity at a given data point x. The noise region is set between −1 and −2 ppm.

SNR measures used in the literature often depend on the spectral processing software used; for example, Bruker's topspin software facilitates computation of SNR via the ‘sino’ command [42, 43, 44]., while Agilent's vnmrj makes use of the ‘dsn’ command [45, 46]. Both compute *signal* as an absolute maximum intensity. A description of these SNR measures, denoted ${\text{SNR}}_{\text{VnmrJ}}$ and ${\text{SNR}}_{\mathrm{Top}.}$, is presented in Section 4 of the SI (Equations (S3) and (S4), respectively).

For the same spectrum, different SNR calculation methodologies can yield substantially different numerical values, potentially affecting the interpretation of detection limits and the comparability of results across studies. Section 4.1 of the SI presents a comparison of the SNR values obtained via the method presented here (${\text{SNR}}_{\mathrm{Int}.}$, Equation (1)) as well as those using commercial software (${\text{SNR}}_{\text{VnmrJ}}$ and ${\text{SNR}}_{\mathrm{Top}.}$). When applied to our mixed metabolite sample spectra, we find the consistent ordering ${\text{SNR}}_{\mathrm{Int}.}<{\text{SNR}}_{\mathrm{Top}.}<{\text{SNR}}_{\text{VnmrJ}}$ across all metabolites and scan numbers. The average ratio of ${\text{SNR}}_{\text{VnmrJ}}/{\text{SNR}}_{\mathrm{Top}.}$ is approximately 2, reflecting a factor of two in their respective noise calculations, while ${\text{SNR}}_{\text{VnmrJ}}/{\text{SNR}}_{\mathrm{Int}.}$ varies between 3 and 5.5 depending on the metabolite, primarily due to our use of a broad integration window that includes baseline contributions.

This has significant practical implications for reported detection limits: for 10 mmol/L glucose in our mixed sample, crossing the conventional LOQ threshold (SNR = 10) requires one, three or five scans when calculated using ${\text{SNR}}_{\text{VnmrJ}},\;{\text{SNR}}_{\mathrm{Top}.}$ or ${\text{SNR}}_{\mathrm{Int}.}$ respectively, with even larger discrepancies observed for lower‐concentration metabolites. The SNR values reported throughout this work should therefore be considered conservative relative to those that would be obtained using commercial software conventions.

## 3 Results and Discussion

In this section, we present results on the acquisition of LF NMR spectra of metabolites at physiological concentrations. Section 3.1 presents analysis of the mixed metabolite sample, exploring the SNR of each metabolite's spectral features as a function of the number of acquisition scans and illustrating how the interpretation of SNR varies depending on the choice of method; Section 3.2 demonstrates how simulation‐based template fitting can be applied to experimental spectra to stabilise the measurement of relative signal intensity as the quality of the overall spectrum degrades; Section 3.3focusses on single‐metabolite samples, evaluating how SNR varies with concentration, pulse sequence and number of scans, and assessing detection limits; Section 3.4 investigates the linearity of the spectrometer's response to increased metabolite concentrations and assesses the extent to which distinct concentration levels can be differentiated at fixed experimental conditions.

### 3.1 Analysis of the Mixed Sample

**FIGURE 2:**
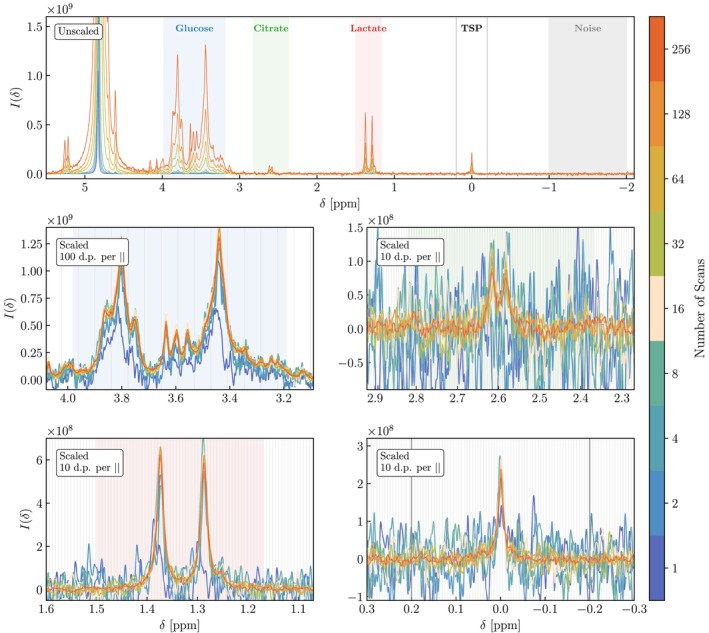
Spectra of a mixed sample containing glucose (10 mmol/L), citrate (0.2 mmol/L) and lactate (2 mmol/L), acquired on a Bruker Fourier 80 spectrometer (80.1 MHz) using a zg30 pulse sequence. The number of scans increases sequentially from $2^{0}$ to $2^{8}$. The top panel displays unscaled spectra across the different number of scans, while the middle and bottom left panels show spectra scaled by the integral of each spectrum's TSP peak, relative to that of the 256‐scan spectrum. Regions shaded blue, green and red denote the ppm ranges used for SNR computation for each metabolite; the noise region is indicated in grey. The region surrounding the TSP peak denoted by vertical black lines indicates the bounds used for Lorentzian fitting during spectral alignment and scaling. For the scaled panels vertical grey lines indicate resolution in chemical shift, where 1 data point (d.p.) spacing is equivalent to $6.1487\times 10^{-4}$ ppm.

We examine the mixed‐metabolite sample, providing an indicative benchmark for assessing how spectral quality evolves with scan number under typical experimental conditions. Figure 2 displays the spectra of the mixed sample acquired with a Fourier 80 spectrometer with a zg30 pulse sequence and a varying number of acquisition scans. The top panel displays the aligned but unscaled spectra across the full relevant spectral range, while the lower panels highlight individual metabolites with intensities scaled to better illustrate the relative levels of signal and noise across the various number of scans. Spectral scaling and centring proceeded as outlined in Section 2 of the ESI, with the 256‐scan spectrum serving as a reference. Although 99.9% 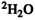 was used as the solvent, the water peak at ∼4.8 ppm remains roughly 200 times higher than the largest metabolite peak in each spectrum, reflecting the relatively high 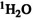 concentration of 55 mmol/L.

At a concentration of 10 mmol/L, glucose exhibits a strong, clearly resolved signal even at low number of scans. In the scaled comparison, glucose peaks are consistent in height and width with the exception of $n_{\text{scans}}=1$, which is affected by a poor TSP fit and subsequent poor scaling and alignment. The glucose SNR increases from 4.3 at a single scan to 68.4 at 256 scans. The ‘Limit of Quantification’ (LOQ) threshold of SNR = 10 [47]. is crossed between four and eight scans, which return values of 8.2 and 14.3, respectively. The 2 mmol/L lactate is visually resolved from $n_{\text{scans}}\geq 2$ with a clear doublet at 1.35 ppm. The SNR increases from 1.6 at one scan to 16.9 at 256 scans. The LOD threshold is exceeded by eight scans, while LOQ is reached at 128 scans.

In contrast, detecting citrate at 0.2 mmol/L proves more challenging. The citrate multiplet at 2.6 ppm remains indistinguishable from baseline noise for $n_{\text{scans}}<64$. At $n_{\text{scans}}=64$, the SNR is 1.8. We estimate that achieving the ‘Limit of Detection’ (LOD) threshold of SNR = 3 would require a doubling of the maximum number of scans here presented. We note that this is likely conservative, as signal is computed as the average absolute intensity over a target region, and the citrate bounds are relatively broad to account for expected secondary peaks at 2.4 and 2.8 ppm. This leads to the inclusion of additional baseline, which therefore worsens the SNR. In Section 4.1 of the SI, we compare this method with routines that instead measure signal as the maximum peak height. Using topspin's routine, we find that SNR > 3 for $n_{\text{scans}}\geq 64$, consistent with visual identification of the citrate multiplet.

These initial results suggest that LF devices are capable of resolving metabolites at mmol/L concentration with relatively few scans. Lower concentrations, however, require longer acquisition times. As discussed, interpretation may depend on the SNR method used; therefore, in the next section, we compare the values obtained using Equation (1) with those from two widely used routines associated with commercial software.

### 3.2 Simulation‐Based Template Fitting

Two major challenges facing commercial in vivo NMR spectroscopy are low‐SNR due to limited acquisition time and the potential for spectral overlap at LF. We present here a proof‐of‐concept method to mitigate both issues: the fitting of simulated metabolite standards to experimental spectra. The data used in this demonstration correspond to the mixed‐metabolite spectra shown in Figure 2. Simulated metabolite standards were generated using CcpNMR's AnalysisAssign. Each synthetic spectrum is comprised of a summed series of Lorentzians, themselves described by spin system matrices. The spectrum is a function described by three free parameters which are optimised to fit experimental spectra: the relative intensity ($A_{\text{rel}}$); the peak width at half maximum intensity (w); and a chemical shift offset ($x_{\text{s}}$). Allowed parameter ranges are unconstrained with the exception of $A_{\text{rel}}\geq 0$, to avoid fitting to negative amplitudes in regions of high stochastic noise. The glucose contribution is modelled as a combination of α‐ and β‐D‐glucose anomers in an assumed 36:64 ratio, as commonly quoted in the literature e.g. [48]; [49]. The fitting routine is available as a plugin for AnalysisAssign at https://github.com/Alex‐Hill94/MetabFit, with further details provided in Section 5 of the SI.

The use of simulation‐assisted spectral analysis within NMR metabolomics has precedent. Commercial packages such as the Chenomx NMR Suite employ libraries of simulated or experimentally derived metabolite signatures to deconvolve complex spectra through template‐fitting approaches [50]. Similarly, automated platforms including Bayesil [51]. and ASICS [52]. fit reference spectra to experimental data to achieve metabolite identification and quantification. Our approach adds to these popular methodologies by exploiting the physics‐based spectral simulation capabilities of CcpNMR AnalysisAssign, which explicitly models spin‐spin coupling effects that become particularly pronounced at low magnetic field strengths. This is especially relevant for benchtop NMR applications, where field‐dependent spectral features differ substantially from HF reference libraries.

**FIGURE 3:**
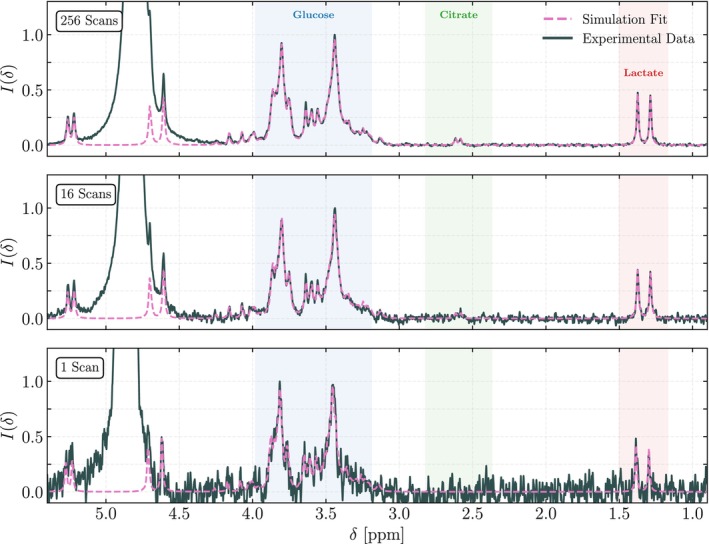
Fitting of simulated metabolite spectra to a mixed metabolite sample containing glucose (10 mmol/L), citrate (0.2 mmol/L) and lactate (2 mmol/L). Spectra were acquired using a Fourier 80 benchtop spectrometer and a zg30 pulse sequence at three representative acquisition settings: 256, 16 and 1 scan(s). Simulated, parameterised templates were generated using spin system matrices and Lorentzian functions in CcpNMR AnalysisAssign, and were optimised to the experimental data using scipy's curve_fit routine. Separate metabolite fits were performed using only data in the shaded spectral regions shown in each panels; the resultant spectra were then summed to produce a composite simulation fit over the full spectral window. Experimental and simulated spectra are normalised to the maximum experimental glucose peak intensity. At 256 scans, strong agreement is observed between the simulation and experiment, including regions outside the fitting windows. At low number of scans, the citrate fitting is either consistent with baseline or appears to fit to noise, in line with earlier findings of SNR ∼ 1 at low metabolite concentration and short acquisition times.

The mixed synthetic spectrum is comprised of a sum of separate fits for each metabolite. Figure 3 shows simulated fits to the experimental spectra for three illustrative cases: 1, 16 and 256 acquisition scans. Amplitudes are scaled to the maximum experimental signal within the 3.19‐ to 3.98‐ppm range, enabling comparison of relative metabolite intensities between experimental and simulated data. Across all numbers of scans, both experimental and fitted spectra show glucose and lactate multiplets with peak heights of approximately 1.0 and 0.5, respectively. There is generally strong overlap between the experimental and fitted spectra, with the exception of citrate at a low number of scans.

While we only consider the lactate signal at around 1.3 ppm during fitting, the relatively weak quartet at 4.1 ppm frequently shows good agreement between simulation and experiment, highlighting the value of fitting only in high SNR regions. The glucose doublet at 5.3 ppm likewise exhibits strong concordance despite not being included in the fitting. The glucose doublet at 4.7 ppm is obscured by the overlapping water peak in the experimental spectra; however, an estimation of its expected intensity may be obtained via the fit. We find that the fitting confidence is higher for the lactate than the glucose, likely a consequence of the simpler nature of its spectral features. For citrate, the fitting results in a positive $A_{\text{rel}}$ for intermediate and high $n_{\text{scans}}$, while the one scan case is effectively flat, consistent with the SNR∼1 for low‐concentration metabolites at short acquisition times.

#### 3.2.1 Analysis of Relative Metabolite Intensities

Both experimental and fitted simulated spectra are impacted by lower SNR. In the former, the noise increasingly impacts the observed signal, and in the latter, the confidence of the fit worsens with a relative increase in noise. We assess which provides the most stable estimation of metabolite signal intensity as the number of scans decreases along with SNR. We measured this as the ratio of the summed signals for pairs of metabolites, expressed as

(2) $${\text{R}}_{XY}=\frac{S_{X}}{S_{Y}}=\frac{\sum_{j=1}^{n}I_{X}(\delta_{j})}{\sum_{i=1}^{m}I_{Y}(\delta_{i})},$$

where X and Y are the two metabolites of interest, containing n and m data points within their allowed chemical shift bounds, respectively. Assessment of signal ratios is relevant in context of personalised health, as the signal is proportional to metabolite concentration, and the ratios of metabolite concentrations are frequently tracked against various health states [53, 54].

**FIGURE 4:**
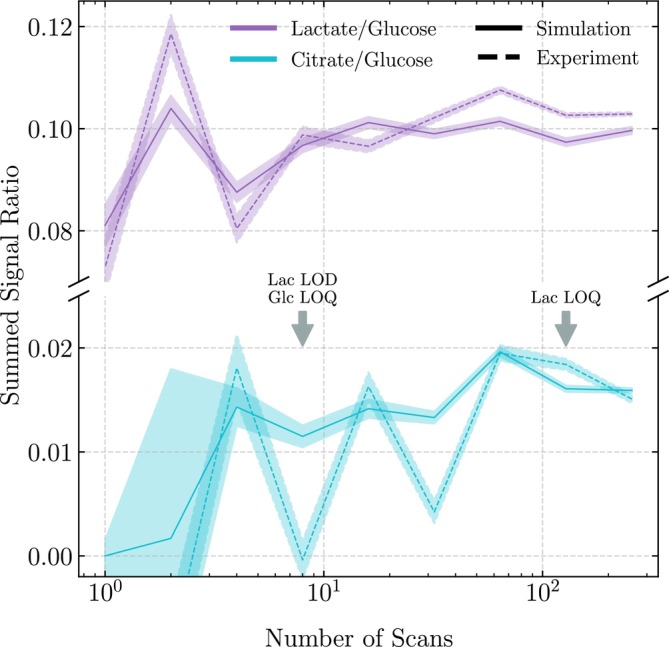
Signal ratios of lactate/glucose and citrate/glucose derived from experimental (dashed lines) and simulation‐fitted (solid lines) spectra, shown as a function of the number of acquisition scans. Ratios use the same pre‐defined metabolite bounds as defined in Section 2.3.1. Shaded regions denote uncertainties, as described in Section 6 of the SI. Approximate limits of detection (LOD) and quantification (LOQ) are indicated for lactate and glucose; citrate never exceeds the LOD threshold. Simulation‐based ratios exhibit greater stability across different numbers of scans, especially in the low‐SNR regime and for citrate in general. Agreement between experimental and simulation ratios at high scan counts supports the validity of the fitting method, while divergences at low scan counts underscore the impact of noise on quantitation and the potential benefits of simulation‐based fitting.

Figure 4 displays the summed signal ratios for lactate/glucose and citrate/glucose as a function of the $n_{\text{scans}}$. Uncertainties on signal ratios were calculated as detailed in Section 6 of the SI. For the maximum 256‐scan experiment, close agreement is observed for the lactate/glucose ratios, with values of 0.103 and 0.100 for the experimental and simulated data, respectively. As $n_{\text{scans}}$ decreases, both datasets deviate from these points; however, the simulation‐based ratios demonstrate greater stability, with a standard deviation of 0.007 as compared with 0.013 for the experimental measurements. Assuming the 256‐scan experiment represents the ground truth, the simulation‐based ratios retain reasonable fidelity down to $n_{\text{scans}}=8$, while for the experimental ratios this is $n_{\text{scans}}=32$.

This distinction between simulation and experiment is more pronounced for the citrate‐glucose ratio. At 256 scans, the experimental and simulated values are again comparable, but both datasets diverge substantially at lower scan counts, with discrepancies exceeding 20% even at 64 scans. The experimental data exhibit a higher standard deviation across all $n_{\text{scans}}$, 0.021 as compared with 0.006 for the simulation‐based ratios; however, we find that this variability is primarily due to the broad spectral region within which the signal intensity of citrate is computed. Reducing the boundaries of citrate region to a third of the size, approximately 1.35σ or 82% of the total signal, reduces the experimental standard deviation to 0.008, while for the simulation, this remains largely unchanged. This highlights the sensitivity of signal measures of noisy experimental data. A further advantage of simulation‐fitting arises in the low‐SNR regime. Because the fit is constrained to known metabolite structures and spectral shapes, it can effectively distinguish signal from noise, defaulting to a baseline fit when no signal is present. This leads to zero‐valued intensities in the absence of confident peak detection, as opposed to experimental integrations which may yield nonzero—or even negative—values due to random noise fluctuations.

Figure 4 shows the number of scans required to cross the LOD and LOQ thresholds for glucose and lactate. Notably, the lactate‐glucose ratio remains stable even below the LOQ of lactate, suggesting that the SNR measure employed here may be conservative for certain applications. In scenarios where the objective is to identify general trends or health deviations rather than exact quantification, simulation‐based ratios may remain meaningful well below formal detection limits. However, because no citrate data meets the LOD threshold, we cannot evaluate whether simulation performance in the sub‐LOD regime reliably reflects what would be expected in high‐SNR data.

### 3.3 Detection of Metabolite Standards at Varying Concentration

A key constraint in the practical implementation rapid metabolite concentration measurement by in vivo MRS, as in development with DigiScan, is the acquisition time required for a robust signal. This depends on both experiment design and metabolite concentration. It is therefore essential to identify which blood metabolites occur at concentrations high enough to be detectable, and which pulse sequences provide the most efficient acquisition.

**FIGURE 5:**
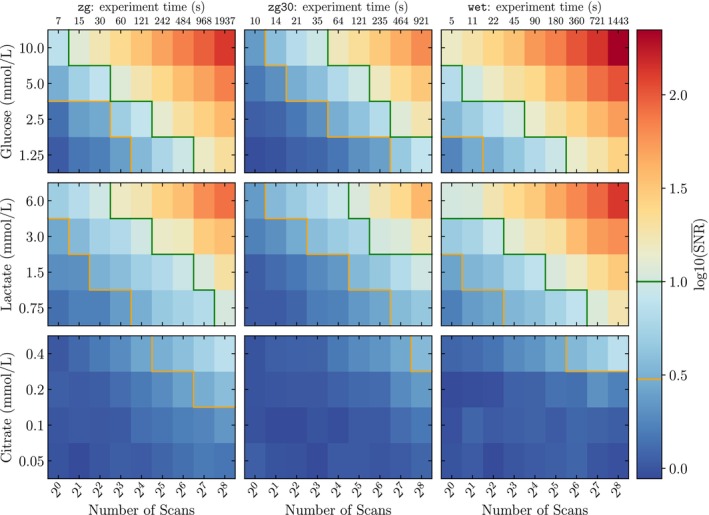
Heatmaps of signal‐to‐noise ratio (SNR) as a function of metabolite concentration, pulse programme and the number of acquisition scans. Rows correspond to different metabolites (glucose, lactate and citrate), while columns represent different pulse programmes (zg, zg30, and wet). The x‐axis shows the number of scans, increasing as powers of 2. The time taken for each experiment is denoted at the top of the figure. Each pixel is coloured according to $\mathrm{lo}g_{\mathrm{10}}(\bar{\text{SNR}})$, representing the mean SNR computed across three samples for a given metabolite concentration and scan count. Orange and green contour lines indicate the conventional SNR thresholds for the Limits of Detection (SNR = 3) and Quantification (SNR = 10), respectively. Data points to the left of these contours fall below the detection or quantification thresholds, while those to the right exceed them. The wet pulse programme achieves the highest SNR for glucose and lactate, whereas for citrate the zg programme is the most effective.

We assessed the capability of a benchtop NMR spectrometer, operating at a field strength comparable to the proposed MRS device, to detect key metabolites at concentrations typical of human blood serum. Spectra of single‐metabolite samples of varying concentration were acquired on a Bruker Fourier 80 using three pulse sequences (zg, zg30, and wet [37, 55].) across a range of scan numbers, allowing evaluation of the SNRs and the respective timings of each concentration‐pulse sequence pairing. These results are shown in Figure 5, and example spectra are displayed in Section 7 of the SI (Figures S3–S5).

As expected, higher metabolite concentration and increased scan number both led to higher SNR for all pulse sequences. At fixed $n_{\text{scans}}$ across all metabolites, zg outperformed zg30. Perhaps counter to expectations, we also found zg30 to be less time‐efficient than zg in returning high SNR.

For glucose and lactate, the water‐suppressing wet sequence yielded the highest SNR under otherwise fixed conditions and was found to be more time‐efficient. This is despite the use of a 99.9% 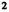
H
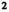
O buffer, which retains a strong residual water peak in zg and zg30 scans. Suppression of this peak, which is approximately 2 orders of magnitude larger than the metabolite signals, allows greater receiver gain, outweighing modest signal loss from the frequency‐selective pulses. With one wet scan (5 s), all but the lowest glucose concentration exhibited SNR > 3, and the highest exhibited SNR > 10. For lactate, eight wet scans are required for all concentrations to exceed SNR > 3 for lactate.

We found that for citrate spectra acquired with wet, only at the highest concentration is SNR > 3 achieved (at 64 scans). In contrast to glucose and lactate, citrate exhibited the highest SNRs with the zg sequence, possibly resulting from wet's frequency‐selective pulses causing a partial signal attenuation, exceeding any SNR gain following reduced interference from the 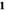
H
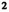
O peak.

Overall for the acquisition settings evaluated, our findings indicate that concentrations above 1 mmol/L are required to rapidly obtain spectra with SNR > 3. This threshold is comfortably met by physiological levels of glucose and lactate across all tested sequences, making them suitable targets for DigiScan and similar applications.

### 3.4 Differentiability of Metabolite Standards at Varying Concentration

Single point‐in‐time metabolite measurements are often insufficient to describe health states, rather repeated measurements are typically required to track metabolic fluctuations. When interpreted in the context of an individual's clinical profile, these variations can provide valuable insights into underlying biochemical and physiological changes. For in vivo MRS trackers to be effective in this context, they must not only detect metabolites but also quantify them or at least reliably distinguish between substantially different concentrations. This requires the system exhibit a predictable response to changes in metabolite concentration, even in short experiments.

**FIGURE 6:**
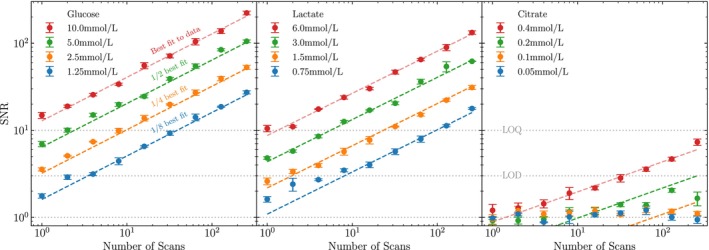
Signal‐to‐noise ratio (SNR) as a function of the number of acquisition scans for different concentrations of glucose, lactate and citrate, measured on a Fourier 80 spectrometer using a wet pulse programme. Metabolite concentrations follow a doubling pattern, as do the number of acquisition scans, which increase from $2^{0}$ to $2^{8}$. Each data point represents the mean SNR computed across three samples per concentration, with error bars indicating the standard error of the mean. Grey horizontal dashed lines indicate the conventional Limits of Detection (LOD, SNR = 3) and Quantification (LOQ, SNR = 10). For each metabolite, a power‐law fit ($\text{SNR}=A\cdot n_{\text{scans}}^{B}$) is applied to the highest concentration data (red points). The fit is then scaled by 1/2, 1/4, and 1/8 to assess the alignment of lower concentrations under the expected proportional relationship.

To appraise the LOD and LOQ of physiological metabolite concentration with the wet pulse sequence and assess their differentiability, we assessed SNR as a function of metabolite concentration and scan number (Figure 6). Equivalent results for the zg and zg30 sequences are provided in Figure S7 (ESI Section 8). Each data point represents the mean SNR across repeated samples, with error bars showing the standard error on the mean.

For glucose SNR approximately doubles for each doubling of concentration for fixed scan number. Lactate follows a similar trend, with the exception of some nonlinearity at low concentration and low scan number. Citrate deviates significantly from this, with all but the highest concentration samples yielding SNRs near one, indicating poor detectability and differentiability between concentrations.

To further quantify these trends, we fit a power‐law model, $\text{SNR}=A\cdot n_{\text{scans}}^{B}$, to the highest‐concentration data for each metabolite. Fits were obtained by linear regression in log‐log space (see Section 8, ESI). For glucose and lactate the fitted exponents are B=0.5 and 0.48, respectively, consistent with the theoretical $\sqrt{n_{\text{scans}}}$ scaling. Citrate yields a lower exponent (B=0.35), but restricting the fit to SNR>3 data increases it to B=0.45, highlighting the domination of noise at low scan numbers.

These findings indicate that physiological concentrations of glucose and lactate yield a predictable, near‐linear response in LF spectrometers, even at low scan numbers. This is illustrated in Figure 6 by rescaling the best‐fit amplitude A from the highest concentration by factors of 1/2, 1/4, and 1/8 to estimate SNR at lower concentrations. For glucose and lactate, these predictions agree with true power law fits to within 10%, with $R^{2}\geq 0.988$ and 0.906, respectively. For citrate, the model fit is reasonable at high concentration ($R^{2}=0.948$), but the extrapolation to lower concentrations fails completely, with $R^{2}<0$.

In practical terms, our results indicate that metabolites at mmol/L concentrations are suitable targets for real‐time in vivo MRS devices such as DigiScan, with concentration differences distinguishable in under 30 seconds. By contrast, citrate's poor performance highlights the limitations of detecting low‐abundance metabolites under these experimental conditions.

## 4 Conclusions

This study demonstrates that LF NMR spectroscopy can rapidly detect and differentiate blood metabolites at physiologically relevant concentrations above 1 mmol/L, establishing the feasibility of benchtop MRS in personalised health applications. Millimolar metabolites such as glucose and lactate are accessible within seconds using water‐suppressing pulse sequences, whereas the acquisition of submillimolar metabolites like citrate remains challenging in short time frames. These findings directly inform the design of in vivo MRS devices such as DigiScan^TM^, clarifying which metabolites are most suitable for real‐time tracking and under what conditions.

We show that the choice of SNR definition can critically affect the classification of spectra relative to defined detection and quantification thresholds, underlining the need for clear reporting and interpretation in LF metabolomics. We also highlight simulation‐assisted spectral fitting as a promising strategy to stabilise quantification in noise‐dominated regimes. This may be particularly significant in a health tracking context where a limited number of targeted metabolites are of interest, reducing the complexity of fitting challenge and potentially extending the practical utility of LF MRS for targeted, real‐time metabolite monitoring.

A key limitation of this study is the use of simplified metabolite standards dissolved in 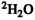. Translation to biological samples such as blood serum will introduce significant challenges, including a dominant 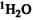 peak, broad lipid and protein signals, and overlapping low‐abundance metabolites. In Section 9 of the SI, we present preliminary results into the analysis of biosamples at low field. Using legacy urine samples, we find that citrate and lactate are readily observable under the same experimental conditions presented in this work. Additional challenges for in vivo spectroscopy include accurate targeting of tissue volume, blood flow dynamics and temperature variability. Future work will address these challenges through optimised acquisition strategies, such as selective excitation and suppression, alongside advanced postprocessing approaches, including template fitting with robust baseline correction and likely the incorporation of machine learning methods. Together, these developments will represent a step towards practical, consumer‐grade MRS devices capable of supporting longitudinal, personalised health monitoring.

## Author Contributions

A.D.H. led the study design and sample preparation, conducted the experimental campaigns, co‐acquired high‐field validation spectra, developed and implemented the simulation‐based fitting methodology, analysed the data and wrote the manuscript. G.T. provided strategic direction for the research, identified target metabolites relevant to commercial device development and contributed to manuscript revision. M.P. supervised experimental protocols, provided training in NMR metabolomics techniques, co‐acquired high‐field validation spectra, contributed metabolomics expertise to data analysis and interpretation and provided text for the methods section. M.H. provided technical support for CcpNMR AnalysisAssign implementation, co‐developed the simulation‐based fitting functions and provided text for the methods section. C.P.W. initiated the collaboration between the parties and secured funding. All authors reviewed and approved the final manuscript.

## Funding

The authors have nothing to report.

## Conflicts of Interest

G.T. is the CEO of ViBo Health Inc., which is developing DigiScan.

## Supporting information



SupplementaryInformation_Updated.pdf.

## Data Availability

Raw data including acquisition and processing parameters are available on the European Bioinformatics Institute metabolomics public repository (Accession number MTBLS6262).

## Endnotes

^1^

https://www.vibo.health/.

^2^
Ratio of 81:19 NaHPO : NaHPO, pH 7.4.
