# Supplementary material for: Detection Limits of Blood Metabolites at Physiological Concentrations Using Benchtop H NMR
Source: NMR Biomed. 2025 Dec 23;39(2):e70215. doi: 10.1002/nbm.70215 (PMC12727471; doi:10.1002/nbm.70215)
Supplement: Supplementary file 1 — SupplementaryInformation_Updated.pdf. [file NBM-39-e70215-s001.pdf]

Electronic Supplementary Material (2025) : 1–15

NMR IN BIOMEDICINE

Electronic Supplementary Material (ESI)

Detection Limits of Blood Metabolites at Physiological Concentrations Using Benchtop ^{1}H NMR

Alexander D. Hill⋆[ab], Gil Travish[c], Marie Phelan[de], Morgan Hayward[f] and Carsten P. Welsch[ab]

[a]Department of Physics, University of Liverpool, Liverpool, United Kingdom.
[b]The Cockcroft Institute, Sci-Tech Daresbury, Warrington, United Kingdom.
[c]ViBo Health Inc, Los Alamos, NM 87544, United States of America.
[d]Department of Biochemistry and Systems Biology, Institute of Systems Molecular and Integrative Biology, University of Liverpool, Liverpool, United Kingdom.
[e]High-field NMR Facility, Liverpool Shared Research Facilities (LIV-SRF), University of Liverpool, Liverpool, United Kingdom.
[f]Department of Molecular and Cell Biology, Leicester Institute of Structural and Chemical Biology, University of Leicester, Henry Wellcome Building, Lancaster Road, Leicester LE1 7HN, United Kingdom.
⋆*a.d.hill@liverpool.ac.uk*.

Contents

1 Validation of metabolite standards 1
2 Spectra acquisition and data processing 2
3 Determination of metabolite chemical shift bounds 3
4 Comparison of common signal to noise measures 4
4.1 Comparison of SNR measures 6
5 Simulation-based template fitting 6
6 Uncertainty estimation for metabolite signal ratios 7
7 Example spectra of single metabolite samples 8
8 Differentiability of metabolites via different pulse sequences 11
9 Analysis of biological sample 13

1. Validation of metabolite standards

The majority of the samples produced in this work were single-metabolite standards at concentrations spanning physiological ranges found in blood. Each metabolite had 12 samples prepared, with three replicates of four concentrations. The metabolites investigated were glucose (1.25, 2.5, 5.0, 10.0 mmol/L), lactate (0.75, 1.5, 3.0, 6.0 mmol/L), and citrate (0.05, 0.1, 0.2, 0.4 mmol/L). The metabolites were chosen due to their wide range of physiological concentrations[1] and association with conditions such as diabetes [2], PTSD [3], and cancer [4]. While high SNR values can be achieved on benchtop devices for metabolites at concentrations in the tens of μmol/L [5], our focus is on what can be detected with a limited number of acquisition scans. The selected metabolites therefore provide a representative range of concentrations to assess the practical capabilities of low-field benchtop NMR, and commercial devices based on similar technology.

To validate the accuracy and consistency of the sample preparation, NMR spectra were acquired for all samples using a Bruker Avance IIIHD 700MHz spectrometer equipped with a TCI cryoprobe. A standard vendor supplied 1D ^{1}H NOESY pulse sequence with presaturation (noesygppr1d) was employed, with 32 scans per sample. Spectral processing was performed using the vendor-supplied automated routine

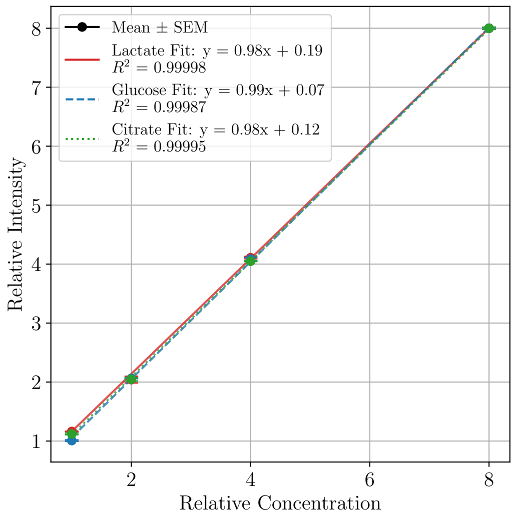

Figure S1: Validation of the single-metabolite sample preparation for glucose, lactate and citrate, as measured by the NMR signal intensity as a function of sample concentration. Units are scaled on each axis such that the maximum concentration and the mean relative intensity is set to eight. Scatter points and error bars denote the mean signal intensity across the three replicate samples, and the standard error on the mean, respectively. Best-fitting lines are found following inverse variance-weighted least squares regression, with optimal parameters and R^2 values displayed in the legend. The strong linearity ($R^2 > 0.9998$) and small errorbars (SEM > 0.057) supports the reliability of the sample preparation protocol.

apk0.noe. The procedure included application of an exponential window function corresponding to a line broadening of 0.3 Hz, Fourier transformation, automated phasing, and spectral alignment to the internal standard, trimethylsilylpropanoic acid (TSP, set at 0.00 ppm). All steps were carried out automatically within the apk0.noe pipeline to ensure consistency and reproducibility across samples.The operational probe temperature was 25°C, which is validated by measuring the distance between peaks of 99.8% Methanol-d4 [6]. Quality assurance of the 3-dimensional shimming is undertaken by asserting that the full width at half max of 2mmol/L sucralose's 0ppm peak is less than 1Hz, following the quality assurance criteria set out by the Metabolomics Standards Initiative (MSI) [7].

Fig. S1 displays the results of the validation, plotting mean signal intensity as a function of concentration with both axes scaled so that the highest concentration and mean signal intensity equal eight. Signal intensities for each metabolite were calculated as a the maximum peak intensity within a corresponding chemical shift range: glucose [3.19, 3.98]; lactate [1.17, 1.50]; and citrate [2.37, 2.82] ppm. See Section 2.3.1 of the main publication for further details. For each metabolite inverse variance-weighted linear regressions were fitted to the data. The calculated high R^2 values indicate that, as expected, the data follows a strong linear relationship between intensity and concentration for each metabolite, supporting the reliability of the sample preparation protocol. The minor deviations from zero intercepts are likely attributable to baseline noise or minor systematic effects. Full spectra parameters and datasets are available via the public repository MetaboLights, at www.ebi.ac.uk/ID-999999.

2. Spectra acquisition and data processing

The Bruker Fourier 80 benchtop spectrometer used in this work operated at a transmitter frequency of 80.1 MHz and a probe temperature of 25°C. Spectra were processed using Bruker's TopSpin software.

The raw Free Induction Decay (FID) signals were multiplied by an exponential line-broadening function, followed by a Fourier transform, and finally an application of the phase parameters present in the dataset, i.e. the 'efp' command. Phase and baseline corrections were undertaken employing the deep learning methodology outlined in Bruderer et al. 2021 [8], i.e. the 'apbk' command.

Each sample contained 100μmol/L TSP, enabling scaling and translation of spectra to enable comparison between measurements. This is achieved through the fitting of a Lorentzian function in the -0.2 to 0.2 ppm region, i.e. about the TSP singlet at ~0.0. The form of the Lorentzian employed is given as

$$\frac{A}{1 + \left(\frac{x - x_0}{\gamma/2}\right)^2}, \tag{S1}$$

where A is the amplitude, γ is the full width at half maximum (FWHM), and x_0 is the centre position. The centre of the returned fit is used to set 0ppm, while the integral of the curve is used to scale the signal intensities when comparing samples. In practice, some low-signal experiments do not result in an optimal fit, in which cases we retain the original spectra for subsequent analysis. We note that scaling does not affect signal-to-noise ratio (SNR) measurements, and any small discrepancies in chemical shift away from expected values are not large enough to make a significant difference.

3. Determination of metabolite chemical shift bounds

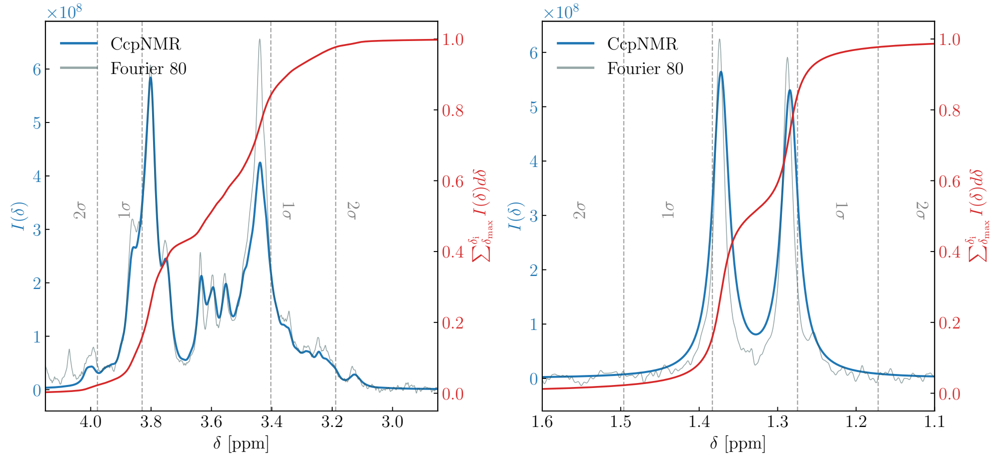

Figure S2: Definition of expected chemical shift bounds for glucose (left) and lactate (right) using simulated spectra from CcpNMR AnalysisAssign [9, 10]. Simulated 80 MHz spectra are shown in blue, with corresponding experimental spectra acquired using a Bruker Fourier 80 benchtop spectrometer overlaid in grey for comparison. The cumulative intensity curves (in red) are normalised and plotted from high to low ppm to identify central peak positions and signal spread. Dashed lines indicate regions containing 1σ (~68%) and 2σ (~95%) of the total signal. We define expected bounds as the region spanning 2σ.

CcpNmr AnalysisAssign [9, 10, 11] is used to provide a theoretical basis for the expected bounds of metabolites. Synthetic spectra are generated for each target metabolite, assuming an 80MHz spectrometer and no noise. For chemical shifts (δ) running from high to low, we compute the running summation of the spectral intensity as

$$S(\delta_i) = \sum_{\delta_j=\delta_{\max}}^{\delta_i} I(\delta_j), \qquad \text{for } \delta_j = [\delta_{\min}, \delta_{\max}] \tag{S2}$$

where $\delta_{\max}$ and $\delta_{\min}$ denote the absolute bounds of the simulated spectral range considered.

Normalising Eq. S2 such that $S(\delta_{\min}) = 1$, we define a metabolite's expected spectral centre as the chemical shift $\delta_{\text{cent.}}$ for which $S(\delta_{\text{cent.}}) = 0.5$. The outer bounds are then set to contain 2σ ($\approx 95.4\%$) of the total spectral intensity. Specifically, the lower and upper bounds $[\delta_{\text{left}}, \delta_{\text{right}}]$ are set such that $S(\delta_{\text{left}}) = 0.0228$ and $S(\delta_{\text{right}}) = 0.977$. As lactate exhibits multiple distinct peak clusters separated by baseline over a large $\Delta\delta$, we restrict our analysis to the prominent cluster at 1.35 ppm.

We calculate definitions of the following chemical shift bounds for each metabolite: glucose [3.19, 3.98] ppm; lactate [1.17, 1.50] ppm; and citrate [2.37, 2.82] ppm. The demonstration of this methodology with respect to glucose and lactate are displayed in Fig. S2. The bounds are used in all subsequent analysis for each metabolite.

4. Comparison of common signal to noise measures

Multiple conventions exist in the literature for the computation of SNR for spectral features. All methods calculate the *signal* within a region associated with the feature of interest, and the *noise* within a broad part of the spectrum absent of any noticeable peaks. However, the means by which the quantities themselves are calculated differ. In this work we compute SNR as the ratio of average intensities in *signal* and *noise* regions, such that

$$\text{SNR}_{\text{Int.}} = \frac{\sum_{j=1}^{n} \text{abs}(I(\delta_j))}{n} \Bigg/ \frac{\sum_{i=1}^{m} \text{abs}(I(\delta_i))}{m}, \tag{S3}$$

where n and m are the number of data-points in the signal and noise regions, respectively, and $I(\delta_x)$ is the signal intensity at a given data point x. We set our noise region to be between -1 and -2ppm.

Calculating *signal* as an average across a range rather than an absolute maximum signal is often preferable when the spectrum under observation is comprised of a cluster closely spaced peaks, or when comparing datasets of different spectral widths. Other common conventions measure signal as the maximal signal intensity, which may be more appropriate in the context of well-isolated and low FWHM peaks, and if the target region contains significant baseline noise. This approach is taken in the popular VnmrJ and TopSpin spectra processing software, developed by Agilent and Bruker, respectively [12, 13, 14, 15].

Agilent's VnmrJ software computes SNR using the 'dsn' command, with the underlying computation

$$\text{SNR}_{\text{VnmrJ}} = \max\left(I(\delta_{i=0}), \ldots, I(\delta_{i=n})\right) \Bigg/ \sqrt{\frac{\sum_{j=1}^{m} I(\delta_j)^2}{m}}, \tag{S4}$$

where the numerator is the maximum peak height in the signal region, and the denominator is the root mean square of intensities in the noise region. Brukers TopSpin software computes SNR via the automated 'sino' routine. The *signal* term is computed in the same manner as in VnmrJ, however the *noise* term is modified such that

$$\text{SNR}_{\text{Top.}} = \max\left(I(\delta_{i=0}), \ldots, I(\delta_{i=n})\right) \Big/ 2 \cdot \mathcal{N}(\delta_i), \tag{S5}$$

$$\mathcal{N}(\delta_i) = \sqrt{\frac{\sum_{i=-n}^{n} I(\delta_i)^2 - \frac{1}{N}\left((\sum_{i=-n}^{n} I(\delta_i))^2 + \frac{3\cdot\left(\sum_{i=1}^{N} i(I(\delta_i)-I(\delta_{-i}))\right)^2}{N^2-1}\right)}{N-1}}. \tag{S6}$$

Here N is the total number of data points in the *noise* region, $n = (N-1)/2$, and $I(\delta_i)$ is the intensity at the i-th point in the noise region. This more complex computation of the noise mitigates the impact of linear drifts in a spectrum's baseline. A comparison of these methods in the context of our work is presented in the main document.

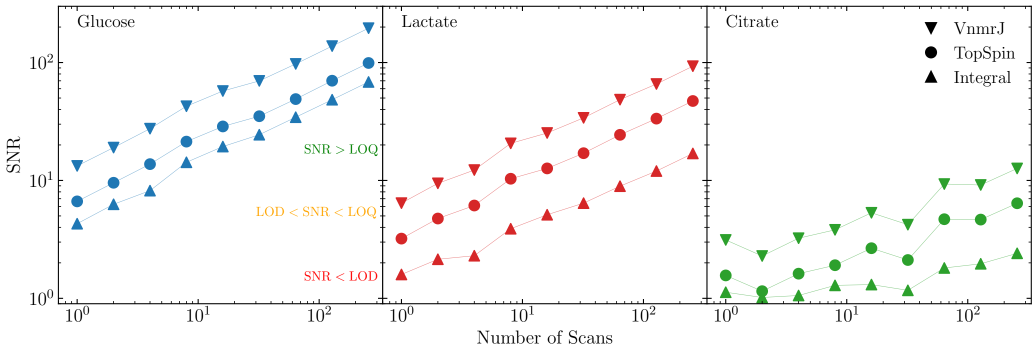

Figure S3: Comparison of three SNR measures applied to spectra of the mixed sample displayed in Fig. 2 of the main document. From left to right, panels display SNR values of glucose, lactate, and citrate, plotted as a function of the number of acquisition scans. Icon shapes distinguish the SNR measures: down-arrows for $\mathrm{SNR}_{\mathrm{VnmrJ}}$, circles for $\mathrm{SNR}_{\mathrm{Top.}}$, and up-arrows for $\mathrm{SNR}_{\mathrm{Int.}}$. Shaded regions and annotations denote the conventional SNR regimes: SNR = 3 corresponds to the 'Limit of Detection' (LOD), and SNR = 10 to the 'Limit of Quantification' (LOQ). A consistent offset is observed, with $\mathrm{SNR}_{\mathrm{Int.}} < \mathrm{SNR}_{\mathrm{Top.}} < \mathrm{SNR}_{\mathrm{VnmrJ}}$. Determination of whether a given metabolite spectrum is above or below the LOQ or LOD can depend on the choice of SNR definition, and $\mathrm{SNR}_{\mathrm{Int.}}$ may be considered conservative when compared with other studies.

4.1. Comparison of SNR measures

Fig. S3 presents the measured SNR as a function of the number of acquisition scans for the spectra of Fig. 2 of the main document. The SNR metric used in this work is given here as $\mathrm{SNR}_{\mathrm{Int.}}$. We compare this approach with two alternatives from popular signal processing software, $\mathrm{SNR}_{\mathrm{Top.}}$ and $\mathrm{SNR}_{\mathrm{VnmrJ}}$, formally defined in Section 4.

For all metabolites and scan counts we observe the consistent ordering: $\mathrm{SNR}_{\mathrm{Int.}} < \mathrm{SNR}_{\mathrm{Top.}} < \mathrm{SNR}_{\mathrm{VnmrJ}}$. The SNR measure used in this work, $\mathrm{SNR}_{\mathrm{Int.}}$, may therefore be considered conservative when compared with other studies.

For each metabolite, the average ratio of $\mathrm{SNR}_{\mathrm{VnmrJ}}/\mathrm{SNR}_{\mathrm{Top.}}$ is 1.99, reflecting the factor of two in the noise expression of Eq. S5 of the ESI. $\mathrm{SNR}_{\mathrm{VnmrJ}}/\mathrm{SNR}_{\mathrm{Int.}}$ varies more widely: for glucose, lactate and citrate the mean ratios are 2.98, 5.07, and 5.50, respectively. This increased variation is primarily a cause of the relative suppression in the measure of the signal term in Eq. S3 through the use of a broad integration window.

This has practical consequences, particularly in quantitative NMR (qNMR) where SNR thresholds can be used to define whether a spectrum is considered reliable for further statistical analysis [16]. In Fig. S3, we illustrate the impact of these definitions by marking the LOD and LOQ regions. For 10 mmol/L glucose, the estimated number of scans required to achieve LOQ is one, three, and five for $\mathrm{SNR}_{\mathrm{VnmrJ}}$, $\mathrm{SNR}_{\mathrm{Top.}}$ and $\mathrm{SNR}_{\mathrm{Int.}}$, respectively. For the lower-concentration metabolites the discrepancies between SNR measures can be even more pronounced, leading to greater uncertainty in identifying the appropriate acquisition regime.

5. Simulation-based template fitting

CcpNmr AnalysisAssign is used to obtain a functional form for the spectrum of a metabolite at a given spectrometer frequency. These functional forms comprise of a series of summed Lorentzians, and are described by three parameters: the relative intensity (A_{rel}), the peak width at half maximum intensity

(w), and a chemical shift offset (x_s). These parameters are optimised to fit to experimental data using SCIPY'S CURVE_FIT.

In Section 3.2 of the main text, we investigate the spectra of the mixed sample containing glucose, lactate, and citrate. Individual metabolite fittings proceed only considering experimental data within their expected bounds. For each metabolite, optimal values of A_{rel}, w and x_s are found, and full-spectral width synthetic data generated. These are summed for a final simulation fit, such that $I_{\text{fit,tot}} = I_{\text{fit,glc}} + I_{\text{fit,lac}} + I_{\text{fit,cit}}$.

To quantify the strength of the total fit we compute the R^2 parameter. We restrict this analysis to between 1.17 and 3.98ppm, the lower and upper bounds of metabolite features at ppm lower than that of water. This enables the quantification of the strength of the fit as a whole, while avoiding biasing from the strong, unfitted water peak. We find that R^2 is proportional to n_{scans}, with $R^2(n_{\text{scans}} = 256) = 0.972$, $R^2(n_{\text{scans}} = 16) = 0.957$, and $R^2(n_{\text{scans}} = 1) = 0.796$. This indicates that for higher n_{scans} the majority of the variance in the signal is explained by the metabolite signals as described by the simulation templates, while at lower n_{scans} there are systematic uncertainties relating to experiment noise.

This method is available as a plugin to CcpNmr AnalysisAssign at github.com/Alex-Hill94/MetabFit.

6. Uncertainty estimation for metabolite signal ratios

Uncertainty in the experimentally derived metabolite ratios R_{XY} (Eq. 2 of the main text) is calculated as

$$U_{XY} = R_{XY}\sqrt{\left(\frac{u_X}{S_X}\right)^2 + \left(\frac{u_Y}{S_Y}\right)^2}. \tag{S7}$$

Here, u_X and u_Y are the uncertainties on the integrated signals for metabolites X and Y, respectively, and for the experimental data are estimated as

$$u_X = n_X \times \sigma_{\text{noise}}, \tag{S8}$$

where n_X is the number of data points within the integration bounds of metabolite X, and σ_{noise} is the standard deviation of the spectrum within a noise-only region, defined here as being between -2 and -1 ppm. This approach assumes Gaussian-distributed noise and uniform uncertainty contribution across the integration region.

For the simulated spectra, the uncertainty estimation accounts for both fit parameter uncertainty and residual variance. The uncertainty u_X metabolite is computed as the quadrature sum of two components,

$$u_X = \sqrt{\sigma_{S_X}^2 + \sigma_{r_X}^2}. \tag{S9}$$

Here, σ_{S_X} is the standard deviation in S_X estimated from 1,000 Monte Carlo simulations. These are generated by resampling the best-fit parameters from a multivariate normal distribution defined by the covariance matrix returned by the fitting algorithm. The parameter σ_{r_X} is the standard deviation of the residuals, defined as the difference between the experimental data and the fitted simulation, over the spectral region associated with metabolite X. Together, these components therefore provide parameter estimation uncertainty and model fidelity in the final reported uncertainty.

7. Example spectra of single metabolite samples

To illustrate the typical quality and characteristics of acquired spectra, representative examples for glucose, lactate, and citrate at a range of concentrations are shown in Figs S4, S5, and S6, respectively. Each spectrum was acquired using 16 scans on a Bruker Fourier 80 spectrometer at 80 MHz. We display spectra resulting from `WET` [17], `zg`, and `zg30` pulse sequences [18]. These examples are intended to provide a sense of signal intensities, noise levels, and the effect of water suppression across the spectra of the single-metabolite standards, which underpin a significant part of the analysis presented in the main document.

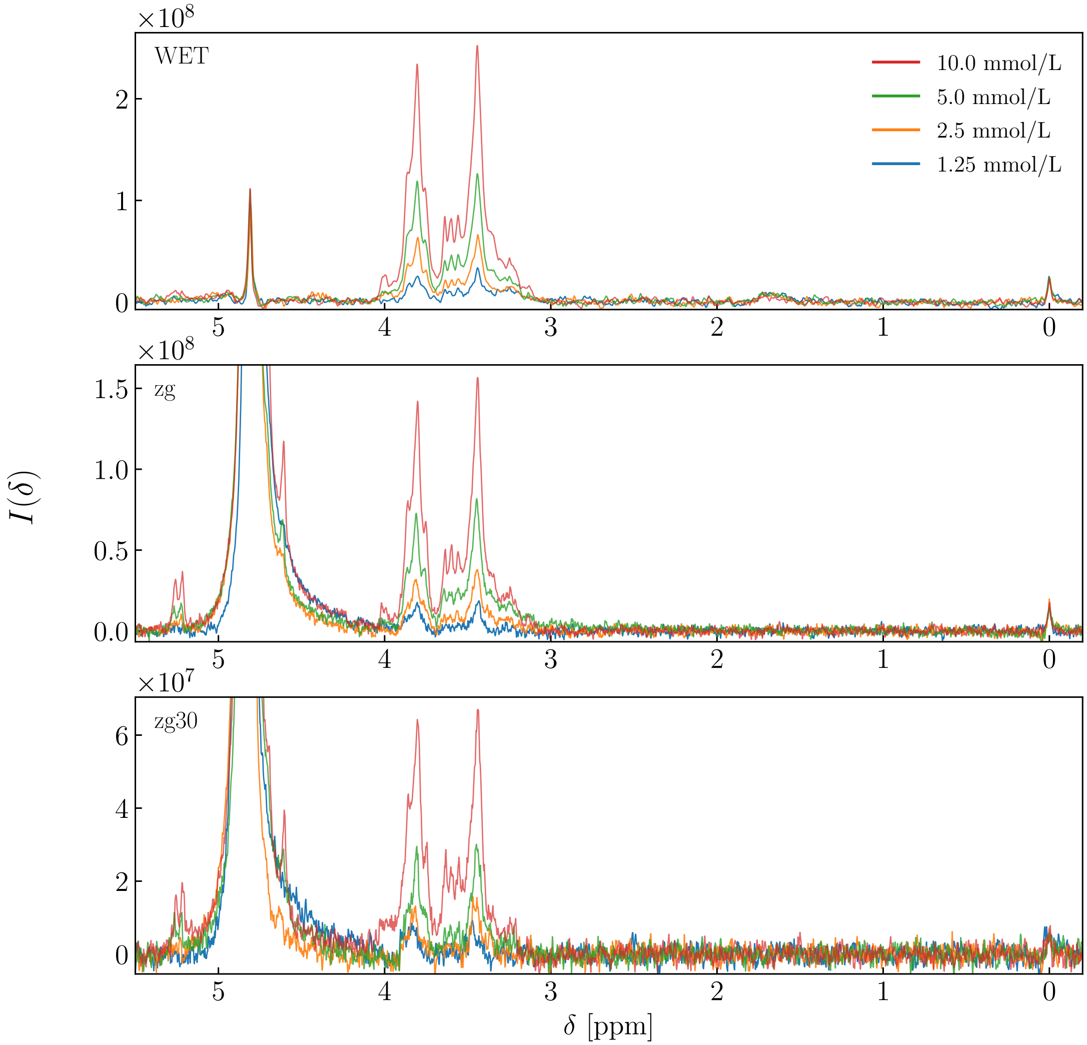

Figure S4: Example NMR spectra of glucose at four concentrations (10.0, 5.0, 2.5, and 1.25 mmol/L), acquired using a Bruker Fourier 80 benchtop spectrometer operating at 80 MHz. All spectra were obtained using 16 acquisition scans. The three panels correspond to different pulse sequences: `WET` (top), `zg` (middle), and `zg30` (bottom). Spectra colour denotes concentration, with higher concentrations yielding stronger signal intensities. The `WET` sequence suppresses the residual water peak, enhancing visibility of metabolite resonances, particularly at lower concentrations. The vertical axis scale is set automatically by TopSpin.

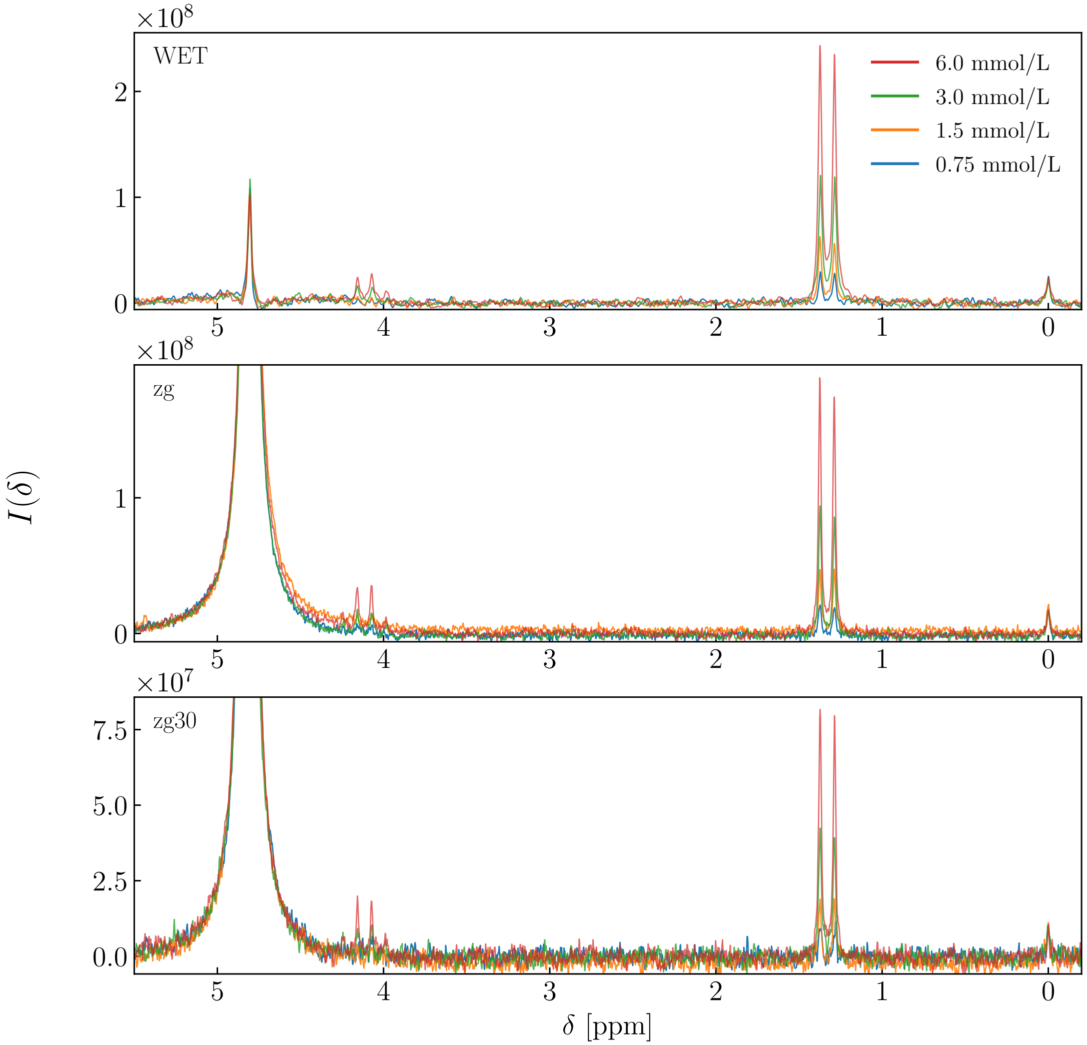

Figure S5: Example NMR spectra of lactate at four concentrations (6.0, 3.0, 1.5, and 0.75 mmol/L). Further details are as described in Fig. S4.

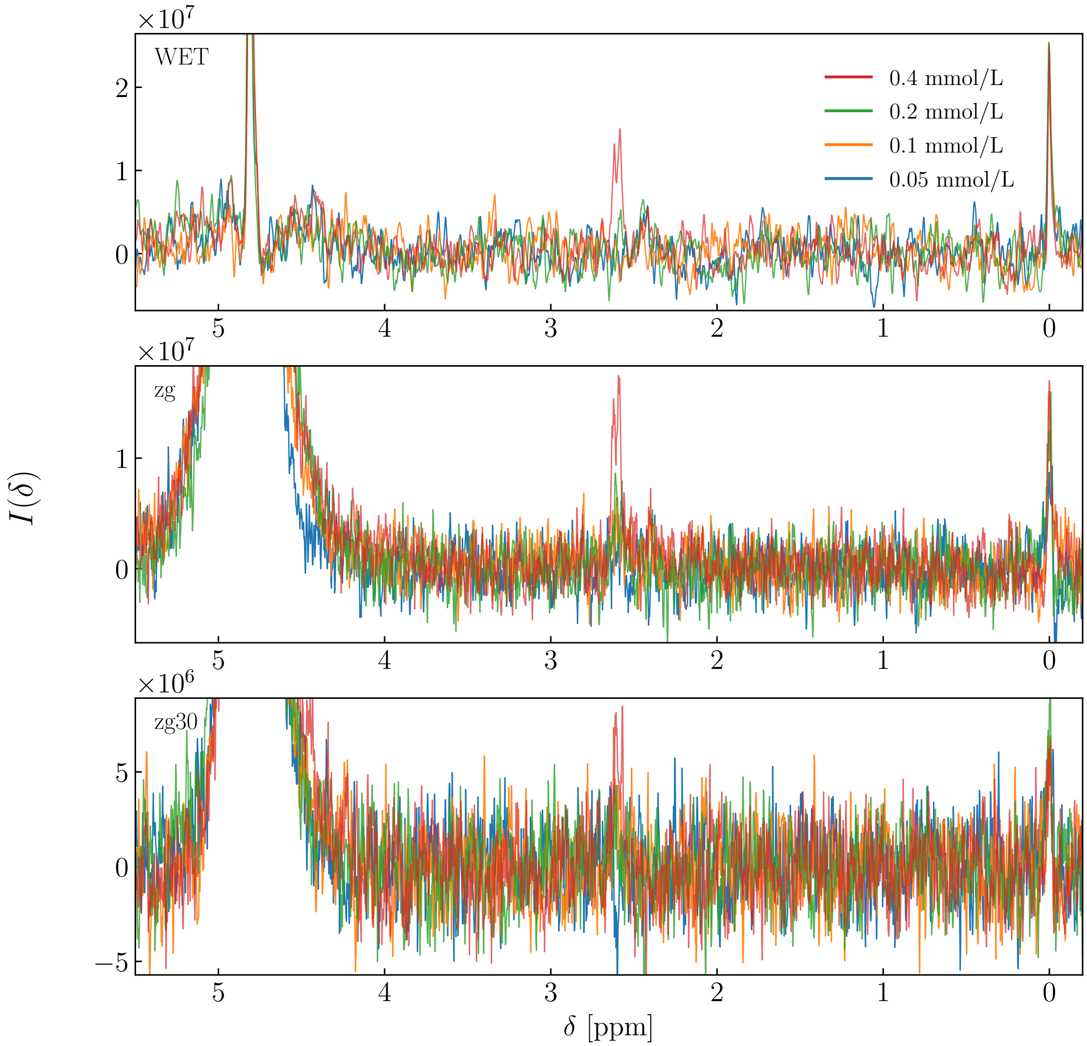

Figure S6: Example NMR spectra of citrate at four concentrations (0.4, 0.2, 0.1, and 0.05 mmol/L). Further details are as described in Fig. S4.

8. Differentiability of metabolites via different pulse sequences

In Fig. S7 and Fig. 6 of the main document, we display SNR as a function of the number of acquisition scans for the `zg`, `zg30`, and `WET` pulse programmes. For each metabolite, we fit a power-law function to the data corresponding to the highest concentration:

$$\mathrm{SNR} = A \cdot n_{\mathrm{scans}}^{B}, \tag{S10}$$

where A and B are parameters to be determined. Since this function is linear in logarithmic space, we perform a linear regression on the transformed variables,

$$\log(\mathrm{SNR}) = \log A + B \log(n_{\mathrm{scans}}), \tag{S11}$$

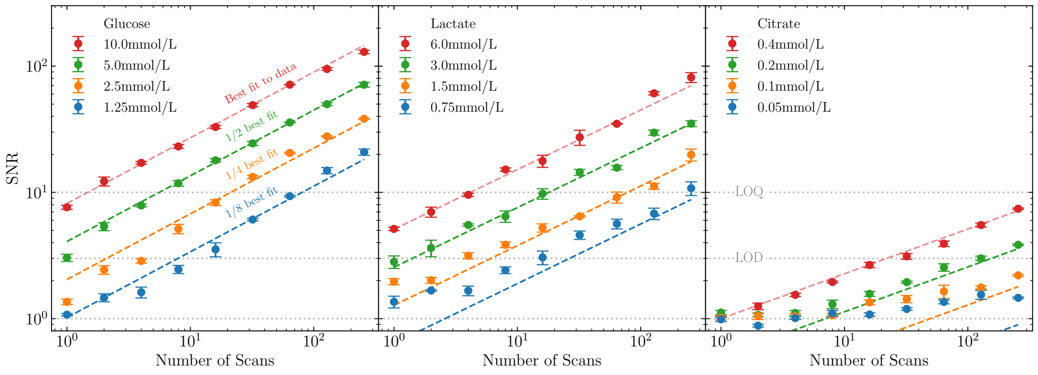

(a)

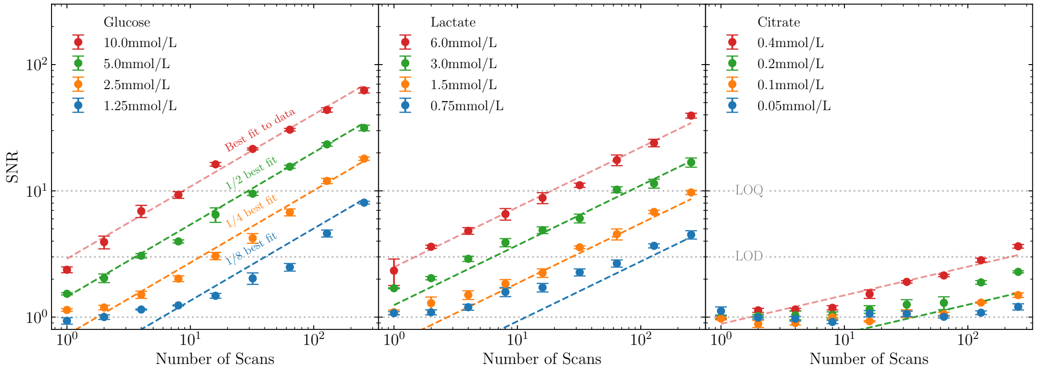

(b)

Figure S7: Signal-to-noise ratio (SNR) as a function of the number of acquisition scans for different concentrations of glucose, lactate, and citrate, measured on a Fourier 80 spectrometer using **(a)** `zg` and **(b)** `zg30` pulse programmes. Metabolite concentrations follow a doubling pattern, as do the number of acquisition scans, which increase from 2^0 to 2^8. Each data point represents the mean SNR computed across three samples per concentration, with error bars indicating the standard error of the mean. Grey horizontal dashed lines indicate the conventional Limits of Detection (LOD, SNR = 3) and Quantification (LOQ, SNR = 10). For each metabolite, a power-law fit ($\mathrm{SNR} = A \cdot n_{\mathrm{scans}}^{B}$) is applied to the highest concentration data (red points). The fit is then scaled by 1/2, 1/4, and 1/8 to assess the alignment of lower concentrations under the expected proportional relationship. For the ZG data, in order of decreasing metabolite concentration, we find $R^2_{\mathrm{Glc.}} = (0.996, 0.988, 0.963, 0.978)$, $R^2_{\mathrm{Lac.}} = (0.989, 0.986, 0.935, 0.645)$, and $R^2_{\mathrm{Cit.}} = (0.991, 0.377, -8.24, -53.6)$. For ZG30, this is $R^2_{\mathrm{Glc.}} = (0.991, 0.993, 0.958, 0.593)$, $R^2_{\mathrm{Lac.}} = (0.992, 0.958, 0.879, -0.643)$, and $R^2_{\mathrm{Cit.}} = (0.941, -1.92, -33.2, -455)$.

using a weighted least squares approach. The weights are taken as the inverse of the variance of $\log(\mathrm{SNR})$, which, assuming small relative errors, is approximated as

$$\sigma_{\log \mathrm{SNR}} \approx \frac{\sigma_{\mathrm{SNR}}}{\mathrm{SNR}}. \tag{S12}$$

Thus, the regression is weighted by $1/\sigma\log(\mathrm{SNR}) = \mathrm{SNR}/\sigma_{\mathrm{SNR}}$, ensuring that data points with lower relative uncertainty contribute more strongly to the fit.

9. Analysis of biological sample

We present a preliminary analysis of metabolite identification in biofluids using low field NMR. A sample consisting of 90% urine and 10% D_2O was measured using a Fourier 80 spectrometer. Urine was selected for practical and ethical reasons, as it was available as a legacy sample from a previous study and represents a readily accessible, low-risk biofluid for method development. Informed consent for this sample was obtained for the stated purpose of aiding NMR metabolomics method development.

Figure S8 displays spectra acquired using a WET pulse sequence on the Fourier 80 device with varying numbers of acquisition scans, compared against a reference spectrum obtained from the same sample using a Bruker Avance IIIHD 700 MHz spectrometer with a 512-scan noesygppr1d sequence. Good spectral agreement is observed at low field for $n_{\text{scans}} \geq 4$, with small molecule metabolites clearly distinguishable. As expected, the 700 MHz spectrum exhibits substantially higher resolution, with numerous well-resolved multiplets, whereas the low-field spectra show broader, overlapping features characteristic of reduced field strength. The slight peak asymmetry in the low field spectra arose from relatively poor shimming during operation.

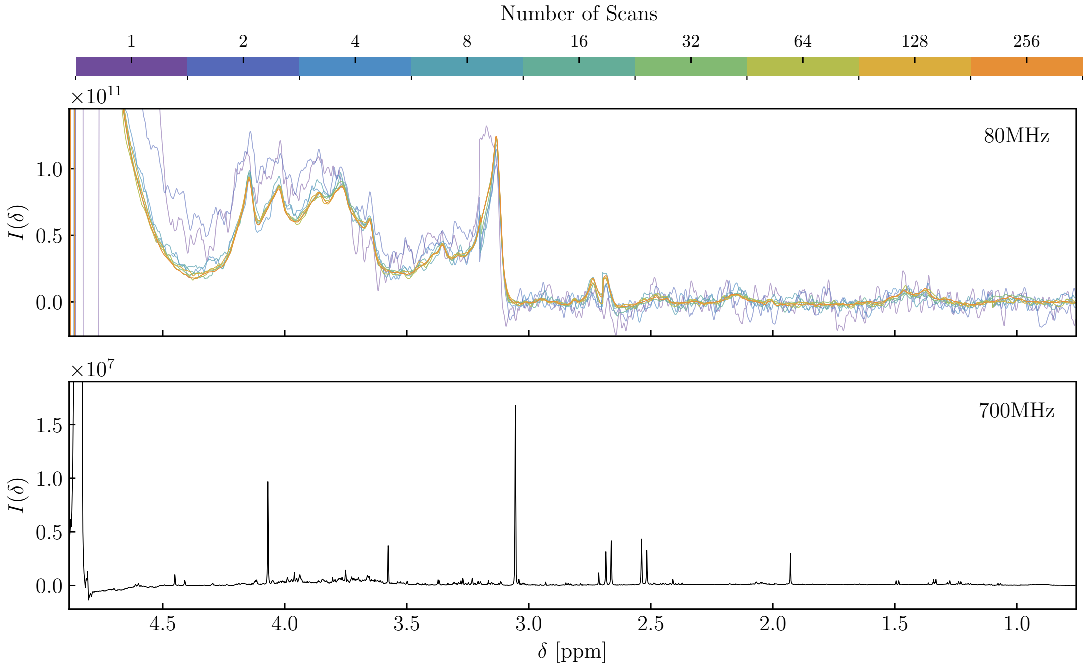

Figure S8: NMR spectra of urine combined with D_2O in a 90:10 ratio. The upper panel shows spectra acquired at low field (80 MHz) using a Fourier 80 spectrometer with a WET pulse sequence, with colour indicating the number of acquisition scans (1–256). The lower panel provides a reference spectrum of the same sample acquired at high field (700 MHz) using a 512-scan noesygppr1d pulse sequence. The high-field spectrum exhibits substantially greater resolution with numerous well-resolved multiplets, whereas low-field spectra show broader features, which are characteristic of a lower magnetic field strength. Metabolite peaks are clearly detectable in low-field spectra.

Figure S9 further demonstrates metabolite detectability by comparing the low-field urine spectrum with individual lactate (6 mmol/L) and citrate (0.4 mmol/L) standards. Spectra are scaled and offset vertically for clarity. After applying a 0.1 ppm chemical shift correction to the urine spectrum, two

prominent doublets align closely with the characteristic lactate and citrate peaks at approximately 1.3 and 2.6 ppm, respectively. Both standard spectra are referenced to TSP at 0 ppm, and the required shift to the urine spectrum likely reflects minor pH or temperature differences between the samples. The additional peaks within the urine spectrum are likely other metabolites commonly found in urine, such as creatinine and various sugars.

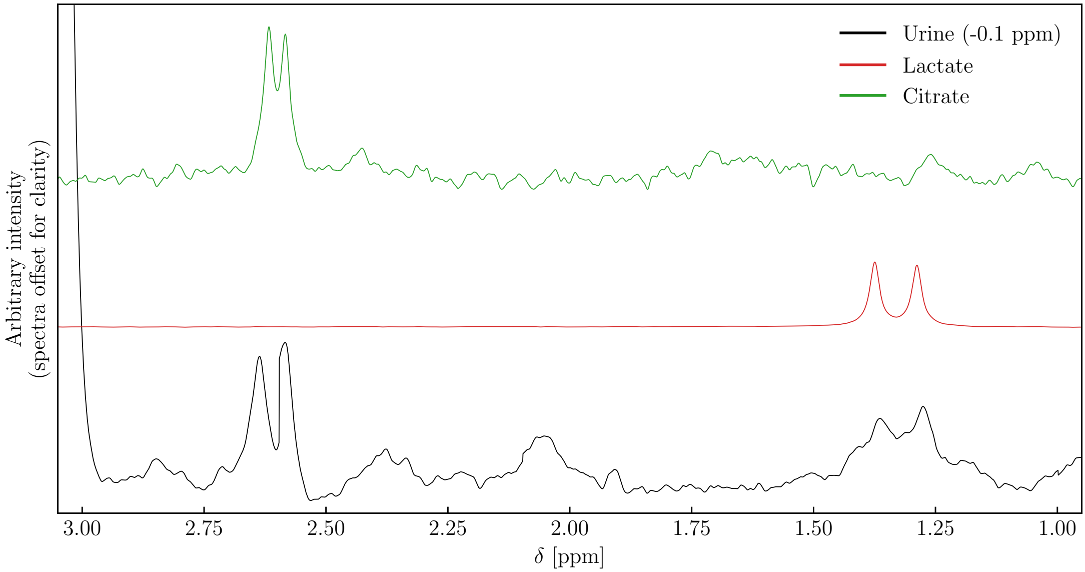

Figure S9: Comparison of urine and single-metabolite standard spectra acquired at low field (80 MHz). The y-axis represents arbitrary intensity with vertical offsets applied for clarity. Urine (black, bottom), lactate (6 mmol/L, red, middle), and citrate (0.4 mmol/L, green, top) spectra were acquired using a 256-scan WET pulse sequence. The urine spectrum has been shifted by –0.1 ppm to account for minor chemical shift differences. Two prominent doublets in the urine spectrum align with the expected positions of lactate (1.3 ppm) and citrate (2.6 ppm), demonstrating that small molecule metabolites are readily identifiable in biological samples at low field.

These preliminary results demonstrate that small molecule metabolites are readily observable in biological samples at low field, supporting the potential of benchtop NMR for biofluid analysis. We acknowledge that extension to blood samples will require additional pulse sequences to mitigate broad signals from proteins and lipids, as well as sophisticated post-processing techniques to maximise the signal obtained from noisy data. Nonetheless, the findings presented here highlight the promise of targeted metabolite detection in biofluids with benchtop NMR.

References

[1] David S Wishart, AnChi Guo, Eponine Oler, Fei Wang, Afia Anjum, Harrison Peters, Raynard Dizon, Zinat Sayeeda, Siyang Tian, Brian L Lee, et al. Hmdb 5.0: the human metabolome database for 2022. *Nucleic acids research*, 50(D1):D622–D631, 2022.

[2] Cristina Valeri, Paolo Pozzilli, and David Leslie. Glucose control in diabetes. *Diabetes/metabolism research and reviews*, 20(S2):S1–S8, 2004.

[3] Synthia H Mellon, F Saverio Bersani, Daniel Lindqvist, Rasha Hammamieh, Duncan Donohue, Kelsey Dean, Marti Jett, Rachel Yehuda, Janine Flory, Victor I Reus, et al. Metabolomic analysis of male combat veterans with post traumatic stress disorder. *PloS one*, 14(3):e0213839, 2019.

[4] R Ravikanth Reddy and Naranamangalam R Jagannathan. Potential of nuclear magnetic resonance metabolomics in the study of prostate cancer. *Indian Journal of Urology*, 38(2):99–109, 2022.

[5] Martin Grootveld, Benita Percival, Miles Gibson, Yasan Osman, Mark Edgar, Marco Molinari, Melissa L Mather, Federico Casanova, and Philippe B Wilson. Progress in low-field benchtop nmr spectroscopy in chemical and biochemical analysis. *Analytica chimica acta*, 1067:11–30, 2019.

[6] M Findeisen, T Brand, and S Berger. A 1h-nmr thermometer suitable for cryoprobes. *Magnetic Resonance in Chemistry*, 45(2):175–178, 2007.

[7] Lloyd W Sumner, Alexander Amberg, Dave Barrett, Michael H Beale, Richard Beger, Clare A Daykin, Teresa W-M Fan, Oliver Fiehn, Royston Goodacre, Julian L Griffin, et al. Proposed minimum reporting standards for chemical analysis: chemical analysis working group (cawg) metabolomics standards initiative (msi). *Metabolomics*, 3(3):211–221, 2007.

[8] Simon Bruderer, Federico Paruzzo, and Christine Bolliger. Deep learning-based phase and baseline correction of 1d 1h nmr spectra. *Public Bruker White Paper*, 2021.

[9] Simon P. Skinner, Rasmus H. Fogh, Wayne Boucher, Timothy J. Ragan, Luca G. Mureddu, and Geerten W. Vuister. Ccpnmr analysisassign: a flexible platform for integrated nmr analysis. *Journal of Biomolecular NMR*, 66(2):111–124, 2016.

[10] Morgan W. Hayward and Geerten W. Vuister. On the importance of open-source databases for nmr-based metabolomics. *Am J Biomed Sci & Res.*, 18(2), 2023.

[11] Hesam Dashti, William M. Westler, Marco Tonelli, Jonathan R. Wedell, John L. Markley, and Hamid R. Eghbalnia. Spin system modeling of nuclear magnetic resonance spectra for applications in metabolomics and small molecule screening. *Analytical Chemistry*, 89(22):12201–12208, 2017. PMID: 29058410.

[12] OpenVnmrJ Community. Openvnmrj: Open source nmr spectroscopy software, 2025. Accessed: 2025-08-19.

[13] Agilent Technologies. *VnmrJ Command and Parameter Reference*, 2025. Accessed: 2025-08-19.

[14] Bruker. Topspin: Nmr data analysis. https://www.bruker.com/en/products-and-solutions/mr/nmr-software/topspin.html. Accessed: 2025-08-19.

[15] Bruker Corporation. *TopSpin User Manual*. UCSB NMR Facility, 2025. Accessed: 2025-08-19.

[16] Torsten Schoenberger, Yulia Monakhova, Dirk Lachenmeier, Stephan Walch, and Thomas Kuballa. Guide to nmr method development and validation – part ii: Multivariate data analysis, 03 2016.

[17] RB Kingsley. Wet, a t1-and b1-insensitive water-suppression method for in vivo localized 1h nmr spectroscopy. *Journal of Magnetic Resonance, Series B*, 104(1):1–10, 1994.

[18] Bruker Corporation. Bruker user library, 2025. Accessed: 2025-08-19.
